# Clinical Trials Required to Assess Potential Benefits and Side Effects of Treatment of Patients With Anorexia Nervosa With Recombinant Human Leptin

**DOI:** 10.3389/fpsyg.2019.00769

**Published:** 2019-05-17

**Authors:** Johannes Hebebrand, Gabriella Milos, Martin Wabitsch, Martin Teufel, Dagmar Führer, Judith Bühlmeier, Lars Libuda, Christine Ludwig, Jochen Antel

**Affiliations:** ^1^Department of Child and Adolescent Psychiatry, University Hospital Essen, University of Duisburg-Essen, Essen, Germany; ^2^Department of Consultation-Liaison Psychiatry and Psychosomatic Medicine, University Hospital of Zürich, Zurich, Switzerland; ^3^Division of Paediatric Endocrinology and Diabetes, Department of Paediatrics and Adolescent Medicine, Ulm University Hospital, Ulm, Germany; ^4^Department of Psychosomatic Medicine, University Hospital Essen, University of Duisburg-Essen, Essen, Germany; ^5^Department of Endocrinology and Metabolism, Medical Center and Central Laboratory, University Hospital Essen, University of Duisburg-Essen, Essen, Germany

**Keywords:** anorexia, leptin, hyperactivity, metreleptin treatment, starvation, physical activity

## Abstract

The core phenotype of anorexia nervosa (AN) comprises the age and stage dependent intertwining of both its primary and secondary (i.e., starvation induced) somatic and mental symptoms. Hypoleptinemia acts as a key trigger for the adaptation to starvation by affecting diverse brain regions including the reward system and by induction of alterations of the hypothalamus-pituitary-“target-organ” axes, e.g., resulting in amenorrhea as a characteristic symptom of AN. Particularly, the rat model *activity-based anorexia* (ABA) convincingly demonstrates the pivotal role of hypoleptinemia in the development of starvation-induced hyperactivity. STAT3 signaling in dopaminergic neurons in the ventral tegmental area (VTA) plays a crucial role in the transmission of the leptin signal in ABA. In patients with AN, an inverted U-shaped relationship has been observed between their serum leptin levels and physical activity. Albeit obese and therewith of a very different phenotype, humans diagnosed with rare congenital leptin deficiency have starvation like symptoms including hypothalamic amenorrhea in females. Over the past 20 years, such patients have been successfully treated with recombinant human (rh) leptin (metreleptin) within a compassionate use program. The extreme hunger of these patients subsides within hours upon initiation of treatment; substantial weight loss and menarche in females ensue after medium term treatment. In contrast, metreleptin had little effect in patients with multifactorial obesity. Small clinical trials have been conducted for hypothalamic amenorrhea and to increase bone mineral density, in which metreleptin proved beneficial. Up to now, metreleptin has not yet been used to treat patients with AN. Metreleptin has been approved by the FDA under strict regulations solely for the treatment of generalized lipodystrophy. The recent approval by the EMA may offer, for the first time, the possibility to treat extremely hyperactive patients with AN off-label. Furthermore, a potential dissection of hypoleptinemia-induced AN symptoms from the primary cognitions and behaviors of these patients could ensue. Accordingly, the aim of this article is to review the current state of the art of leptin in relation to AN to provide the theoretical basis for the initiation of clinical trials for treatment of this eating disorder.

## Introduction

Improvements in the treatment of patients with AN have been made over the past decades. In particular, patients have profited from an increased awareness of the disorder both among the lay public and health care professionals leading to earlier treatment and a change in the perception of the disorder (Hebebrand et al., 2004; Hebebrand and Bulik, 2011; American Psychiatric Association [APA], 2013). Importantly, the DSM-IV A criterion (American Psychiatric Association [APA], 2000) “refusal to maintain body weight at or above a minimally normal weight for age and height” was replaced with “restriction of energy intake relative to requirements” in DSM-5, thus correcting the long-standing notion of an actively and willfully pursued reduced food intake (see Hebebrand and Bulik, 2011). As such, more rigid treatment regimens including for instance extended periods of bed rest and therapeutic cutback or even prohibition of exercise have given way to a more fluid approach toward these patients acknowledging the fact that as yet unknown mechanisms prevent the maintenance of a healthy body weight. The shift in the perception of this eating disorder was undoubtedly supported by the discovery of the complex pathways underlying body weight regulation and the insight that, for example, only a low percentage of individuals with obesity can successfully maintain a reduced body weight over time (Friedman, 2004; Wing and Phelan, 2005; Bray and Wadden, 2015;MacLean et al., 2015; Soleymani et al., 2016).

Despite these improvements, AN remains a debilitating disorder that substantially perturbs somatic and psychological maturation during the important developmental stages of adolescence and early adulthood. In those two-thirds of patients in whom recovery eventually occurs, the mean duration of AN and subsequent eating disorders amounted to 7.5 (±4.7) years according to a Swedish study (Wentz et al., 2001). Furthermore, recovery in itself is relative because former patients may continue to have residual eating disorder-related cognitions and behaviors at the subclinical level (Espindola and Blay, 2013). Noteworthy, AN is the mental disorder with the highest standardized mortality rate (Sullivan, 1995; Arcelus et al., 2011). Accordingly, both psychotherapeutic and pharmacological treatments of AN still leave substantial room for improvement (Hebebrand et al., 2019, in press).

Whereas today body weight fear and body image disturbances are viewed as essential diagnostic criteria for AN (DSM-5), we need to appreciate that the combination of underweight and amenorrhea was common in times prior to the coining of the term AN by Gull (1997). Thus, chlorosis was a disorder of females that persisted in different forms from the 16th century to approximately 1920; descriptions of chlorosis refer to amenorrhea and a “capricious or depraved appetite,” which included “anorexia, or at least a reduced food intake, sometimes associated with nausea and vomiting” and pica (Loudon, 1980). The phenomenon of the transient occurrence of fasting saints in the middle ages (Vandereycken and Van Deth, 2001), too, raises the question of a many centuries old core pathology that includes underweight to the degree of starvation.

Our longstanding interest in leptin and AN began with the first study to demonstrate that serum concentrations in patients with AN are reduced (Hebebrand et al., 1995) published one year after the discovery of leptin (Zhang et al., 1994). We later for the first time showed that treatment of food restricted rats with leptin prevents the development of semi-SIH in a rat model for AN (Exner et al., 2000) similar to the model ABA (Routtenberg and Kuznesof, 1967). In this combined selective review and medical hypothesis article, we focus on the possibility to reduce starvation-induced symptoms in patients with AN by treatment with metreleptin (a human recombinant leptin analog).

We initially provide the reader with a synopsis of the function of leptin and the development of metreleptin, its success in treatment of patients with congenital leptin deficiency, and its approval for the treatment of generalized lipodystrophy, which as in AN is metabolically characterized by hypoleptinemia. We continue by distinguishing between potential somatic and psychological/behavioral benefits that we would expect after initiation of metreleptin treatment of patients with AN based on the alterations incurred during starvation. Because two controlled trials (Welt et al., 2004; Sienkiewicz et al., 2011) have already been conducted in patients with hypothalamic amenorrhea – albeit without a current diagnosis of an eating disorder – we cursorily review these studies. We extend our previous major focus on the potential to treat the hyperactivity of patients (Hebebrand and Albayrak, 2012) in light of the aforementioned rodent data. Due to the relevance of the reward system for both AN and leptin, we selectively summarize the current research status. We argue for the potential of metreleptin to curtail the reward inherent to both the characteristic dieting behavior and hyperactivity of these patients, and thus, facilitation of weight restoration. We additionally explore the possibility that application of metreleptin at an early stage of AN may prevent the progression to an addictive like disease state. We conclude by delineating target symptoms for future clinical studies and by pointing out potential adverse events that may result from metreleptin treatment. In light of the dire need to increase treatment options for patients with AN, we hope that our admittedly selective compilation of research findings will nevertheless help to initiate clinical trials examining effects of metreleptin treatment in AN.

## Leptin: Function, Regulation, and Recombinant Leptin

Leptin is involved in numerous biological functions, which apart from weight regulation include innate and adaptive immunity, reproduction, and bone formation (Peelman et al., 2014). The hormone leptin is comprised of 167 amino acids (Halaas et al., 1995; Auwerx and Staels, 1998), which in its non-glycosylated mature form is reduced to 146 amino acids (Peelman et al., 2014). As an adipokine, it is expressed mainly in adipocytes (Kawakami et al., 1999). Upon secretion into the blood stream, leptin is largely bound to the short form of the LEPR, which lacks the transmembrane domain (Chan et al., 2002); its circulating concentration is influenced by gender, adiposity, sex hormones, and recombinant human (rh) leptin administration (Chan et al., 2002). In lean persons with 21% or less body fat, 60–98% of total leptin was bound whereas in obese individuals, more than 50% of the total leptin concentration circulates in the free form (Sinha et al., 1996). Circulating plasma leptin levels increase in proportion to body fat stores (Fried et al., 2000). Expression is elevated by feeding [about 4–7 h postprandial (Fried et al., 2000), increased insulin (Wabitsch et al., 1996; Marques-Oliveira et al., 2018), and glucocorticoids (Wabitsch et al., 1996; Fried et al., 2000)]. Androgens contribute to gender differences in leptin production and inhibit leptin expression in human adipocytes (Wabitsch et al., 1997). Leptin concentrations dropped by approximately 75% in females who underwent a 60 h fast (Bergendahl et al., 1999). Leptin is predominantly actively transported across the blood–brain barrier by means of a complex saturable transport system (Banks et al., 1996; Pan and Kastin, 2007; Kastin and Pan, 2016) including the soluble form of the LEPR (Kastin and Pan, 2000, 2008). Only a small proportion of leptin reaches the brain by passive diffusion (Diaz et al., 2006). Leptin can finally reach all regions of the brain, not only the hypothalamus (Banks et al., 2000).

Six LEPR isoforms have been identified. As delineated above, the short form of the LEPR is relevant for the systemic availablity of the hormone (Peelman et al., 2014). Only the LRb encompassing 1162 residues including an intracellular domain of 302 amino acids is presumed to be capable of JAK/signal-transducer activator of transcription 3 (STAT3) signaling. This isoform is highly expressed in the specific nuclei of the hypothalamus relevant for body weight regulation, in other brain regions (Mercer et al., 1996), and in many peripheral tissues including, for instance, the reproduction system (ovaries, uterus), lung, bone, blood, tongue, and hair follicles (Hoggard et al., 1997; Iguchi et al., 2001; Bjorbaek and Kahn, 2004). The extent to which central or peripheral binding of leptin is relevant for its specific functions is still a matter of research (Margetic et al., 2002; Peelman et al., 2014). The functions of the other isoforms have not yet been clarified; they are assumed to play a role in the transport across the blood–brain barrier and in renal clearance of leptin.

**FIGURE 1:**
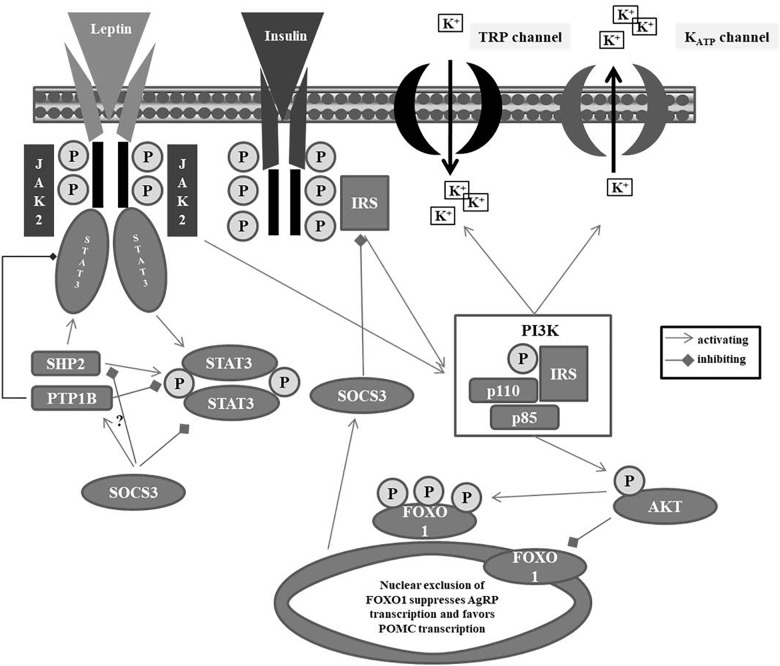
Leptin metabolism pathway. IRS: insulin receptor substrate; JAK: Janus kinase; KATP: ATP-sensitive potassium channel; FOXO1: Forkhead box protein O1; PI3K: phosphatidylinositide 3-kinase; PTB1B: protein tyrosine phosphatase 1B; SHP2: Src homology phosphatase 2; SOCS3: suppressor of cytokine signaling 3; STAT3: signal-transducer activator transcription 3; TRP: transient receptor potential.

Activation of the LRb (Tartaglia et al., 1995), a type I cytokine receptor encoded by *LEPR* (Tartaglia et al., 1995), initiates the JAK – (STAT3) pathway (Fernandes et al., 2015) (see Figure 1). After binding to hypothalamic neurons, autophosphorylation of JAK2 and subsequent recruitment and phosphorylation of STAT3 take place. Phosphorylated STAT3 thereafter dimerizes; the dimer is able to enter the nucleus, where it binds to specific DNA elements in the *POMC* or *AgRP* promoter regions in the hypothalamus causing *POMC* activation (Ernst et al., 2009) and *AgRP* repression (Varela and Horvath, 2012). Subsequent to activation of the STAT3 pathway, leptin induces SOCS3 (suppression of cytokine signaling 3), which functions as a feedback inhibitor of leptin signaling (Bjorbaek et al., 1999). Leptin sensitivity within this signaling cascade is further controlled by (a) PTP1B, which dephosphorylates STAT3, thus, reducing leptin sensitivity of expression of *POMC* (Banno et al., 2010) and (b) the SH2 domain-containing protein tyrosine phosphatase-2 (SHP2), which also dephosphorylates STAT3 at different positions (Banno et al., 2010) to maintain leptin sensitivity. SHP2 is thought to integrate leptin and estrogen signals in females (He et al., 2012). Last but not least, SOCS3 inhibits the actions of SHP2 to repress the phosphorylated pSTAT3 pathway adding to the complex orchestrated interplay in leptin signaling pathways (St-Pierre and Tremblay, 2012).

In a neuron specific manner, leptin is also capable to activate the PI3K pathway thereby leading to (a) a depolarization of POMC neurons via downstream non-specific cation TRP channel activation and (b) hyperpolarization of AgRP neurons by PI3K-mediated opening of K_ATP_ channels resulting in a potassium outflow (Gavello et al., 2016). In contrast to leptin, insulin hyperpolarizes both POMC and AgRP neurons by PI3K-mediated activation of KATP channels (Qiu et al., 2014; Huang and Xiao, 2018). Downstream of PI3K, insulin and leptin both stimulate the phosphorylation and nuclear exclusion of Forkhead protein (FOXO1), thus suppressing AgRP transcription and enabling POMC transcription (Kitamura et al., 2006). The increments of POMC and after its cleavage of α-, ß-, and γ-MSH increase the inhibitory tone in the melanocortin system and result in reduced appetite (Raab et al., 2003; Horvath et al., 2010; Hinney et al., 2013, 2014; Lucas et al., 2015).

There is clear evidence that leptin is involved – in concert with insulin – in midbrain reward circuits (Fulton et al., 2000; Davis et al., 2010; Hebebrand et al., 2014; Coccurello and Maccarrone, 2018). Thus, receptors for insulin and leptin are expressed on dopaminergic (DA) neurons in the VTA/substantia nigra (Hommel et al., 2006) as shown immunohistochemically (Figlewicz et al., 2003), hence substantiating that these metabolic hormones have the capacity to reach the respective brain areas (Banks et al., 2000) and alter reward behaviors and motivational drives for food intake (Figlewicz, 2016). LEPR binding neurons are, however, absent in the amygdala and striatum, but the central amygdala receives extensive projections from LEPR binding DA neurons originating in the VTA (Leshan et al., 2010). Animal models revealed that at five stimulation sites near the fornix, food restriction enhanced the effectiveness of rewarding electrical stimulation; the rewarding effect was attenuated by ICV infusion of leptin (Fulton et al., 2000). Interestingly, the stimulation of some neighboring sites was insensitive to food restriction but enhanced by central leptin administration pointing toward a not yet fully understood Janus effect of leptin regarding the reward system (Fulton et al., 2000). Leptin signaling in the VTA is also involved in the regulation of anxiety-related behaviors (Liu et al., 2011, 2015).

### History of the Development of Metreleptin and Its Use as a Drug

In 1995, Amgen Inc. (Thousand Oaks, CA, United States) bought the commercial rights on metreleptin from Rockefeller University and involved scientists (Fliesler, 2009). At that time, hopes were erroneously high that metreleptin would prove successful for treatment of multifactorial obesity (Rosenbaum et al., 2008) and obese patients with type II diabetes (Moon et al., 2011). In 2006, the rights were sold to Amylin Pharmaceuticals (subsidiary of Bristol-Myers Squibb since 2012), where leptin was tested in combination with its diabetes drug pramlintide (Fliesler, 2009; Chou and Perry, 2013; FDA, 2014). However, despite favorable weight-loss data (Ravussin et al., 2009), Amylin Pharmaceuticals discontinued further development due to “commercial considerations” (FDA, 2014). AstraZeneca, through its partnership with Bristol-Myers Squibb, acquired the global rights for metreleptin in February 2014 and sold it to Aegerion in January 2015 (AstraZeneca, 2014).

Metreleptin with the brand name Myalept^®^ was first approved in 2014 by the FDA and indicated to treat the complications of leptin deficiency in patients with congenital or acquired generalized lipodystrophy after submission of a biologics license application by Amylin Pharmaceuticals (FDA, 2014; Meehan et al., 2016). In July 2018, the European Medical Agency (EMA, 2018) also granted marketing authorization to Aegerion for Myalapta^®^ for treatment of lipodystrophy (confirmed congenital generalized or acquired generalized, or familial or acquired partial lipodystrophy) (orphan decision number: EU/3/12/1022; EMA, 2018).

The human hormone leptin is instable *in vivo* (Roujeau et al., 2014) with a short plasma half-life of mean 24.9 ± 4.4 min (Klein et al., 1996). Metreleptin (r-Met-hu-leptin) is a non-glycosylated 16 kDa recombinant analog with one additional methionine residue at the amino terminus (half-life of 3.8–4.7 h) developed by Amgen for subcutaneous administration (McLennan et al., 2003) and tested in clinical trials for obesity (Heymsfield et al., 1999). After five trials, results were disillusioning. Obese individuals presented with high circulating leptin levels; an elevation of these levels failed to induce adequate weight loss during recombinant leptin therapy (FDA, 2014), thus supporting the concept of “leptin resistance.”

Metreleptin represents a mechanistically based targeted therapy for individuals with rare mutations of the leptin gene entailing inborn functional leptin deficiency (Farooqi et al., 1999). It is one of very few causal treatments currently available for monogenic types of obesity (Farooqi et al., 1999; Farooqi and O’Rahilly, 2014; Kuhnen et al., 2016; Muller et al., 2016; Collet et al., 2017; Clement et al., 2018). Aside from an immediate reduction of appetite/hunger and subsequent medium-term substantial weight loss, the treatment with recombinant leptin entails also positive effects on fertility, cognition, and mental health-related aspects (Paz-Filho et al., 2008) presumably triggered by intra- and extra-hypothalamic effects of leptin in the brain (Paz-Filho et al., 2011; Farooqi and O’Rahilly, 2014). Similar to the *ob/ob* mouse (Chehab et al., 1996), human inborn leptin deficiency results in hypogonadotropic hypogonadism with an impact on the maturation of the reproductive system in affected girls. Treatment with (r-metHuLeptin) leads to a rapid induction of gonadotropin secretion and menarche in pubertal and post-pubertal females (Montague et al., 1997; Farooqi et al., 1999; Bluher et al., 2009; von Schnurbein et al., 2012).

## Adaptation to Starvation and the Key Role of Hypoleptinemia as the Major Endocrine Trigger

A prolonged negative energy balance (i.e., reduced energy intake relative to energy expenditure) leads to starvation and triggers complex neural, metabolic, hormonal, and behavioral adaptations to promote survival. The maintenance of energy supply for the brain and the protection of lean mass are crucial within this adaptation process (Ahima and Flier, 2000a). Carbohydrate metabolism is switched to fat-based metabolism via a fall in insulin and rise in counter-regulatory hormones, i.e., glucagon, epinephrine, and glucocorticoids. A decrease in thyroid and gonadal hormones, increased adrenal glucocorticoids, decreased body temperature, and increased appetite represent further adaptive processes, whose net effect is to stimulate gluconeogenesis to provide glucose for vital cellular function and supply fatty acids for use by skeletal muscle. Importantly, energy utilization is minimized during fasting via suppression of thyroid thermogenesis and curtailment of procreation and growth (Ahima and Flier, 2000b).

Keys et al. (1963) extensively studied the somatic and psychological effects of starvation (see Table 1) in the Minnesota Starvation Experiment, during which young male adults were food restricted (mean energy intake of ∼1600 kcal/day taking individual metabolic needs into account) over a period of 6 months, until they reached a body weight corresponding to approximately 75% of baseline weight. The effects of starvation have also been analyzed in areas of famine and include studies on prenatal and intergenerational as well as long-term somatic and mental effects (e.g., Dutch famine; Susser and Stein, 1994; Susser et al., 1998; Painter et al., 2005). Because of the associated nutrient deficiencies, these results are not directly transferable to AN. Most patients with AN do not initially develop profound nutrient deficiencies (Van Binsbergen et al., 1988) because of comparatively high nutrient-density of their energy restricted diet.

**Table 1:** Starvation in humans: somatic and mental/behavioral symptoms (Michaels, 2013; Gale Encyclopedia of Medicine, 2019).

Somatic symptoms	Mental/behavioral symptoms
Shrinkage of organs and gradual loss of their functions:	Hunger, craving, pre-occupation with food
Gastrointestinal tract	
Heart	
Lungs	
Liver	
Kidneys	
Ovaries or testes	
Chronic diarrhea	Abnormal eating behavior
	Eating smallest amounts
	Eating extremely slowly
	Ritualized eating behavior
Anemia	Depressed mood
Reduction in muscle mass and consequent weakness	Anxiety
Lowered body temperature combined with extreme sensitivity to cold	Irritability
Decreased ability to digest food because of lack of digestive acid production	Inflexible thinking
Immune deficiency	Limited spontaneity
Swelling from fluid under the skin	Restrained initiative
Delayed puberty	Restrained emotional expression
Amenorrhea	Social withdrawal
Bradycardia and arrhythmia	Loss of ambition
Hypoglycemia and abnormal glucose tolerance	Rigidity
Protein deficiency	Reduced cognitive ability and memory
Osteoporosis	Impaired concentration
Dry and discolored skin, lanugo hair	Decreased sex drive


Despite the obvious importance of starvation in the phenotype of AN (Hebebrand and Bulik, 2011) DSM-5 merely provides recommendations for the definition of a “significantly low body weight in the context of age, sex, developmental trajectory, and physical health” including the use of a BMI cut-off of 18.5 kg/m^2^ in adults and the fifth BMI age centile in children and adolescents with AN. The DSM-5 reference to physical health seemingly suggests that somatic symptoms of starvation should have set in to endorse the weight criterion and at the same time negates the fact that mental health is also affected by starvation.

Leptin acts as one, if not the key endocrine trigger, of the adaptation processes required to enhance the likelihood of survival during starvation (Ahima and Flier, 2000b). As fat mass decreases during a period of a negative energy balance, circulating leptin levels drop below critical thresholds, thus, initiating cascades affecting the step by step adaptation to starvation. In parallel to the reduced secretion of leptin upon food restriction both the hypothalamic (Giovambattista et al., 2000) and peripheral LEPRs are upregulated (Cohen et al., 2005), indicating an elevated sensitivity to the hormone during starvation.

**FIGURE 2:**
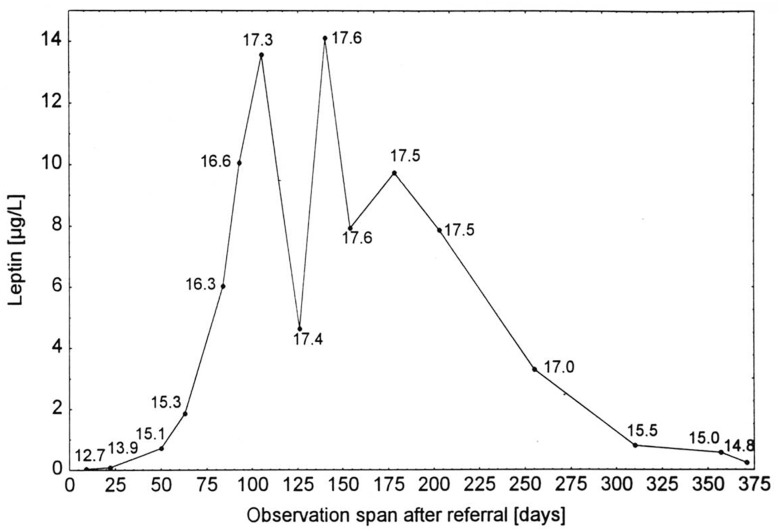
Serum leptin concentrations of a patient with anorexia nervosa (Hebebrand et al., 1997). Serum leptin concentrations of a patient with anorexia nervosa at admission for inpatient treatment, during weight gain, intermittent weight maintenance, and renewed weight loss (numbers indicate BMI in kg/m^2^; Hebebrand et al., 1997). The figure is reproduced with the permission of the copyright holder (Prof. Dr. Johannes Hebebrand).

Patients with AN present hypoleptinemia in accordance with both their reduced energy intake and fat mass (Hebebrand et al., 1995, 1997, 2007; Grinspoon et al., 1996). Consistent with results obtained in normal weight and overweight individuals, serum leptin levels in patients with AN show a higher correlation to body fat than to BMI (Grinspoon et al., 1996; Mathiak et al., 1999). The serum levels are below those of age, BMI, and sex matched controls including constitutionally thin females (Hebebrand et al., 1997; Eckert et al., 1998; Kopp et al., 1998; Germain et al., 2007; Föcker et al., 2011). A serum leptin level of approximately 2 μg/L predicts both AN and a lifetime history of secondary amenorrhea in underweight females and also largely separates patients with AN from those with bulimia nervosa (Kopp et al., 1998; Föcker et al., 2011), indicating that serum leptin can be used to screen for AN or serve as a diagnostic marker (Hebebrand and Bulik, 2011). Weight restoration expectedly entails an increase in leptin secretion, which after several weeks can intermittently exceed that of BMI and sex matched healthy controls (Figure 2).

Similar to individuals with inborn functional leptin deficiency, the reduced function of the reproductive axis and amenorrhea in AN has been linked to the hypoleptinemia of patients with AN (Hebebrand et al., 2003, 2007; Hebebrand and Albayrak, 2012). Mean serum log10 leptin levels over the first 4 weeks of inpatient treatment were correlated with mean FSH, LH, and estradiol levels, respectively (Kopp et al., 1997, 1998). Upon weight restoration, the rise in leptin secretion precedes that of LH and FSH (Ballauff et al., 1999; Wabitsch et al., 2001).

## Proven Efficacy of Recombinant Leptin for Treatment of Hypothalamic Amenorrhea and Bone Loss and Its Potential for Starvation-Related Reduced Blood Cell Production

The fact that the application of recombinant leptin in eight patients over a 3-month period led to menstruation and/or growth of follicles in excessively exercising and/or low weight females who presented with hypothalamic amenorrhea of at least a half year duration, can be viewed as proof of the crucial role of leptin in reproductive function (Welt et al., 2004); within the small scaled RCT improvements of reproductive function were not observed in the six controls. Metreleptin treatment increased mean LH levels and LH pulse frequency after 2 weeks and increased maximal follicular diameter, the number of dominant follicles, ovarian volume, and estradiol levels during the 3-month long treatment period. Three and two of the eight patients had an ovulatory menstrual cycle or a pre-ovulatory follicular development and withdrawal bleeding during treatment. Levels of free triiodothyronine, free thyroxine, insulin-like growth factor 1, insulin-like growth factor-binding protein 3, bone alkaline phosphatase, and osteocalcin all increased significantly in the eight treated females (Welt et al., 2004). Whereas acute eating disorders represented an exclusion criterion in this study, it is unknown if one or more of the eight treated females had a positive history for an eating disorder and AN in particular.

Partially as a result of hypoleptinemia, adolescent patients with AN show poor bone accrual followed by increased bone loss, which can entail lifelong low bone density, degraded bone architecture, and a higher risk of fractures (Sienkiewicz et al., 2011; Chou and Mantzoros, 2018). Again as a proof of principle, treatment with recombinant leptin of eleven lean and strenuously exercising hypoleptinemic females with hypothalamic amenorrhea over a period of nine months within a RCT increased bone mineral content and as a trend bone mineral density in comparison to the nine controls who received placebo (Sienkiewicz et al., 2011).

Based on leptin’s stimulating effects on hematopoiesis (Cioffi et al., 1996; Gainsford et al., 1996), it can be speculated that recombinant leptin may prove beneficial in starvation-related severe reduced blood cell production, which represents an infrequent somatic symptom among patients with AN. In a sample of 318 patients ascertained between 1991 and 2012, 17% of patients had anemia, 8% neutropenia, and 9% thrombocytopenia. These hematologic abnormalities were associated with the duration of illness and protein energy malnutrition including BMI (De Filippo et al., 2016). Substantially higher rates have been observed in a more severely emaciated adult sample; weight gain, achieved via short term hospitalization, substantially reduced the rate of neutropenia (Sabel et al., 2013). The hematologic deficiencies observed in patients with AN have been attributed to starvation-mediated gelatinous marrow transformation which resolves with nutritional rehabilitation (Sabel et al., 2013). Single case reports of patients with AN have shown that life-threatening sepsis may ensue as a consequence of neutropenia (Foppiani et al., 2014; Komatsu et al., 2018). In conclusion, in the proposed future clinical trials (see below), we recommend to co-assess hematological parameters to determine if metreleptin improves the hematological status of patients with AN.

## Behavioral and Cognitive Symptoms of Anorexia Nervosa Potentially Amenable to Treatment with Metreleptin

Apart from a potentially beneficial effect of metreleptin on starvation-related somatic symptoms, we perceive the need to assess its effects on behavior and cognition of patients with AN in future clinical trials. We perceive severe hyperactivity as the currently primary potential indication for treatment of patients with AN with metreleptin; however, we subsequently also address potentially beneficial effects on cognitions and the reward system.

### Is Hyperactivity in Anorexia Nervosa Amenable to Treatment with Metreleptin?

Severe hyperactivity presents a substantial challenge to the patient, caregivers, and treatment teams (Casper, 2018). In light of rodent studies that have convincingly demonstrated that SIH can be successfully suppressed and treated via exogenous application of leptin, we summarize both the rodent data and the clinical phenotype in humans including studies looking into an association between leptin levels and physical activity levels in patients with AN.

### Activity-Based Anorexia (ABA) as a Rodent Model for Anorexia Nervosa

Food restriction to one hour per day in rats or 2–4 h per day in mice entails increased RWA and a decline in food intake entailing weight loss, which can exceed 30% of baseline weight (Hall and Hanford, 1954; Routtenberg and Kuznesof, 1967; Ross et al., 2016). In addition to the classical model of ABA, the initiation of a food restriction to 60% of *ad libitum* intake termed semi-SIH also results in increased RWA by 300–400% within days (Exner et al., 2000). Immediately prior to the daily presentation of food in ABA, rodents will further increase their activity (food-anticipatory activity). Whereas an increased level of activity is usually compensated by an elevated energy intake, both ABA and SIH can ensue in the death of the rodent due to starvation (Routtenberg and Kuznesof, 1967). In contrast, rats in cages without a running wheel survive the restricted access to food. Accordingly, it is potentially only the combination of timed availability/shortage of food and access to running wheels that initiates the precipitous reduction in body weight (Foldi et al., 2017a).

Activity-based anorexia (ABA) recapitulates many of the pathophysiological and behavioral hallmarks of AN, including a reduction in food intake, excessive exercise, dramatic weight loss, loss of reproductive cycles, hypothermia, and anhedonia (Foldi et al., 2017a). ABA also captures the hypoleptinemia-induced up- or downregulation of the hypothalamus-pituitary-end organ axes entailing hypercortisolemia, reduced levels of FSH and LH along with other endocrine alterations including hyperghrelinemia, and physiological changes such as hypothermia (Hebebrand et al., 2003; Ross et al., 2016). As pointed out by Foldi et al. (2017a), the major obstacle in relation to AN is the utilization of a model that incorporates “voluntary” food restriction rather than imposed starvation. Because rats in the ABA model do not eat sufficiently during the 60–90 min *ad libitum* access to food to survive (in contrast to control rats without a running wheel in their cage subjected to the same temporal restriction of food intake), this “voluntary” reduction in food intake may appear more similar to the AN phenotype in humans than the SIH model with forced energy restriction. However, the overall similarity in symptoms between ABA and SIH suggests that this aspect is of minor importance.

The elevated levels of physical activity have been interpreted in evolutionary terms as “displaced food-foraging behavior” akin to times of food shortage (Sodersten et al., 2008). An alternative hypothesis focuses on the prevention of hypothermia (Gutierrez and Vazquez, 2001). In the ABA model, provision (Sherwin, 1998) of a voluntary access to a warm plate prevented the development of hypothermia and reduced, but did not abolish RWA (Hillebrand et al., 2005a, 2008). Accordingly, hypothermia may increase hyperactivity, but does not solely trigger this behavior. Nevertheless, data derived from the ABA model have led to the proposition that increasing ambient temperature may prove beneficial to patients with AN (Hillebrand et al., 2005a; Carrera and Gutierrez, 2018). Further discussion is, however, out of the scope of this review and will be covered in another contribution to this special issue. Interestingly, Foldi et al. (2017b) showed that an activation of the dopamine system substantially increased food intake and prevented weight loss in the ABA model, but left the locomotor activity unchanged.

**FIGURE 3:**
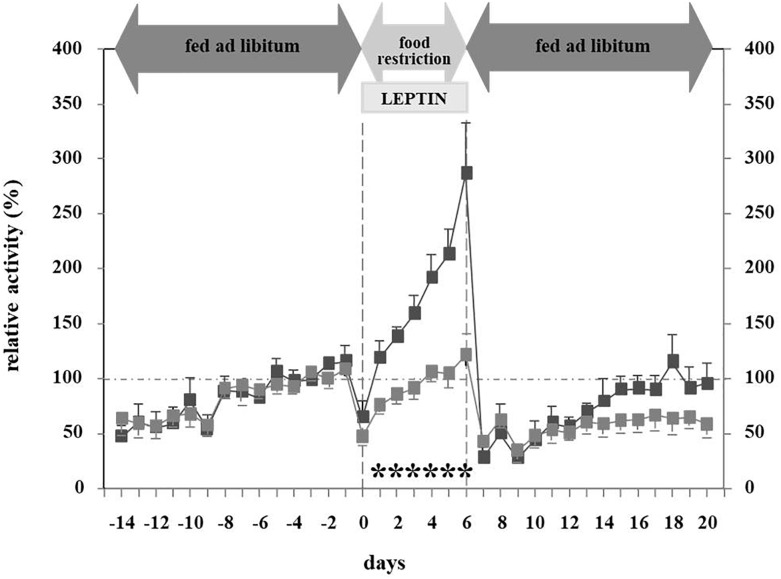
Relative activity at different feeding states and leptin application (Exner et al., 2000). The figure is reproduced with the permission of the copyright holder (Prof. Dr. Johannes Hebebrand).

**FIGURE 4:**
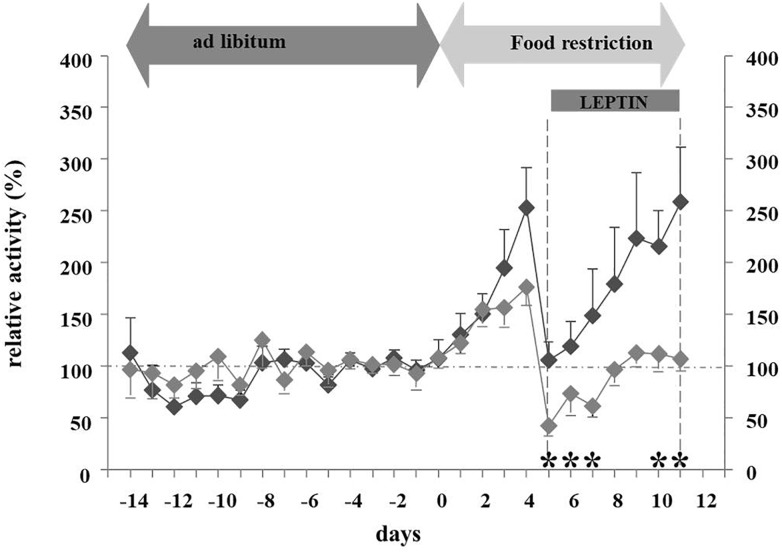
Mechanism and pathways linking food starvation, leptin, and physical activity according to a semi-starvation-induced hyperactivity rodent model (adapted after Exner et al., 2000). Upper part of the figure displays the pathway of rodents exposed to food starvation with a subsequent loss in body weight due to elevated physical activity. The lower part of the figure displays the pathway of rodents who first developed SIH and were then treated with leptin via a mini-pump. Here, physical activity levels reached baseline physical activity level (after implementation).

In light of leptin’s role in the adaptation to starvation (Ahima and Flier, 2000b), we hypothesized that hypoleptinemia may trigger the hyperactivity in SIH (Exner et al., 2000). Indeed, the subcutaneous implantation of a mini-pump releasing recombinant leptin (31 μg/day) over a period of 7 days concomitantly to the initiation of food restriction completely suppressed the development of hyperactivity in male rats, whereas those treated with vehicle increased their RWA by 300–400% (Figures 3, 4). Furthermore, implantation of the mini-pumps 5 days after the initiation of food restriction successfully reduced RWA to baseline values in those rats which received leptin in contrast to those that were treated with vehicle; suggesting for the first time that recombinant leptin can be used to treat hyperactivity in patients with AN (Exner et al., 2000). Surprisingly, despite the large difference in locomotor activity, hyperactive controls did not lose more body weight during semi-starvation for one week than leptin-treated rats, suggesting that leptin treatment had increased the resting metabolic rate.

Hillebrand et al. (2005b) extended these findings to the ABA model. ICV leptin treatment (4 μg/day) decreased RWA both during the light and dark phases in female rats. Only the vehicle-treated ABA rats developed hyperactivity and food-anticipatory activity in the hours before feeding. Activity levels were not influenced by leptin in *ad libitum* fed rats. Furthermore, food-restricted sedentary rats did not reduce activity when treated with leptin, indicating potential specificity of the effect of leptin for ABA. The investigators postulated that leptin reduces the rewarding properties of RWA (Hillebrand et al., 2005b). Leptin treatment decreased food intake in all conditions (*ad libitum*–feeding, food-restricted sedentary rats, ABA), but most strongly in the ABA model, thus indicating that this treatment has strong effects on food intake which override the homeostatic drive to eat. The development of hypothermia was prevented during the initial 4 days of the experiment in the leptin treated animals. The effects of the reduced energy expenditure as a result of relative hypoactivity were outweighed by leptin-induced reduced energy intake and relative hyperthermia. After 4 days, the relative body weight was not significantly different between leptin and vehicle treated rats (76 and 81% of baseline body weight).

**FIGURE 5:**
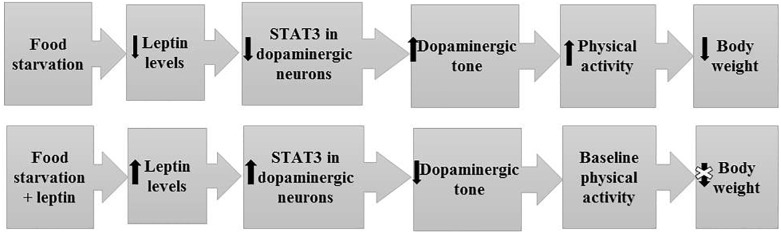
Model for the effect of metreleptin treatment on the dopaminergic tone, physical activity and body weight.

More recently, rodent studies have focused on the underlying mechanisms and pathways. Verhagen et al. (2011) demonstrated that acute bilateral leptin injection in the VTA decreased RWA in a dose-dependent manner. Because the application of the dopamine receptor antagonist flupenthixol also reduced activity levels in the ABA model (Verhagen et al., 2009), a leptin-induced reduction of DA tone via LEPRs in dopamine neurons of the VTA was postulated (Hommel et al., 2006; Liu et al., 2011). Fernandes et al. (2015) indeed found that the DA tone in the VTA is influenced by leptin via STAT3, thus affecting activity levels and running rewards. STAT3-knockout in DA neurons (STAT3DAT KO) resulted in substantial increases in both spontaneous activity and endurance running of the respective mice. Both control and STAT3DAT KO received either saline or recombinant leptin intra VTA prior to a conditioned place preference task. While leptin-treated controls showed suppressed running reward behavior, leptin had no decreasing effect on running reward in STAT3DAT KO mice. Since a viral-mediated restoration of STAT3 in mice reversed the phenotype of the STAT3DAT KO and leptin showed no effect on running reward behavior in the knockout model, leptin seemingly inhibits running rewarding effects through STAT3 in VTA dopamine neurons (Figure 5). Evidence was found for dopamine-opioid interactions in the nucleus accumbens that may contribute to heightened running reward. Because STAT3 loss of function had little influence on the anorectic actions of leptin, hedonic, or compulsive feeding behavior, the involvement of STAT3 signaling in DA neurons was seemingly specific in the control of physical activity. Thus, endurance training may be intrinsically rewarding via moderation of LepR-STAT3 signaling in DA neurons. The investigators speculate that the rewarding properties of physical activity may promote the runners high, excessive exercise and addictive like compulsive behaviors in prone individuals. In light of the difficulties with respect to the dissection of the cognitive and behavioral mechanisms involved in elevated activity levels in patients with AN (Holtkamp et al., 2006; Sternheim et al., 2015), it is worthwhile to point out that selective deletion of the LEPR in dopamine neurons produces anxiogenic-like behavior and increases DA activity in amygdala (Liu et al., 2011).

### Hyperactivity in Anorexia Nervosa

An elevated level of physical activity subsequently referred to as hyperactivity or motor activation, to include mild forms, has early on been recognized as a key symptom of patients with AN (Bruch, 1962; Crisp, 1968; Gull, 1997); “for it seemed hardly possible that a body so wasted could undergo the exercise which seemed agreeable” (Gull, 1997). According to Bruch (Bruch, 1962), “overactivity is rarely spontaneously mentioned by the patient – certainly not in long-standing cases – but it can be recognized with great regularity if looked for. Sometimes there has been an intensified interest in athletics and sports; more often, these activities appear to be aimless, e.g., walking by the mile, chinning and bending exercises, or just refusing to sit down or literally running around in circles.” Bruch observed a cessation of “overactivity,” if patients achieved an advanced degree of emaciation. Even if the amount of exercise did not consistently appear large to Bruch, she expressed amazement in light of the “lacking awareness of fatigue and weakness corresponding to the stage of malnutrition. On the contrary, the subjective feeling is that of not being tired and of wanting to do things.” Patients have been found to value “improving tone” as important and health and enjoyment as less important reasons to exercise (Keyes et al., 2015).

Motor activation includes fidgeting, frequent performance of both isotonic and isometric (maintenance of an active posture) muscle contractions to excessive exercise and sport activities (Alberti et al., 2013). Whereas outpatients, for example, jog, swim, or cycle daily for extended time periods, inpatients may constantly walk around the ward, move their extremities while sitting, maintain postures requiring isometric muscle contractions, or engage in push-ups or other exercises in their rooms, particularly when they feel unobserved. In an unspecified fraction of patients, hyperactivity can represent a truly acute or chronic debilitating symptom of their eating disorder.

The range of lifetime prevalence rates for “excessive physical activity” in patients with AN varies between 38 and 80% (Davis et al., 1997), albeit with large inter-individual qualitative and quantitative differences (Alberti et al., 2013). In a review of “compulsive exercise” in adolescent AN, an even wider range between 16 and 83% was reported. Clear-cut hyperactivity (Fietz et al., 2014) may wax and wane within an individual patient, with the majority reporting that physical activity levels steadily increased during the period when food intake decreased the most (Davis et al., 1994). Because systematic medium or long-term prospective longitudinal data are scarce – many studies have relied on recalled data – the proportion of patients who intermittently experience such mild or severe hyperactivity cannot reliably be specified. According to a study that followed up 37 adolescent patients for 1 year during treatment (Kostrzewa et al., 2013), physical activity levels were found to decrease in the subgroup of 11 patients with initially high levels of physical activity and vice versa to increase in those 26 patients with initially low physical activity, so that in the end, both subgroups had comparable levels. Physical activity remained stable after 1 year. Recovered patients with initially high physical activity had a significantly higher fat mass during the follow-up (Kostrzewa et al., 2013); unfortunately, leptin levels were not measured at admission. On a cross-sectional basis, 35% of 153 consecutively admitted adult inpatients (mean age 26.0 ± 8 years; mean BMI 15.0 ± 1.7 kg/m^2^) were classified as excessive exercisers, defined via a minimum of 1 hour of obligatory exercise performed for at least 6 days per week in the month preceding admission (Bewell-Weiss and Carter, 2010). Based on our own clinical experience, we estimate that 10–15 and 30–50% of adolescent and adult patients, respectively, are hyperactive to an extent that the treatment team attempts to curtail this behavior.

Methodological differences in measurement, inpatient or outpatient settings, length of observation, age, illness duration, and mean BMI have entailed heterogeneous results. Methods to measure physical activity have included retrospective analysis of medical records, activity diaries, experience sampling, questionnaires or self-ratings using visual analogue scales, expert ratings using semi-structured interviews, clinician rated scales of physical activity and motor restlessness, and devices such as actometers and pedometers to measure movement (Hebebrand et al., 2003; van Elburg et al., 2007; Gummer et al., 2015). Patients seemingly under-report their physical activity upon comparison with actometry-based data (Alberti et al., 2013). In an adolescent inpatient setting, the ratings of physical activity based on visual analogue scales filled in by two nurses were reasonably well correlated with average actometer activity scores (*r* = 0.61) for the 18 patients (van Elburg et al., 2007). In contrast, the correlation between the patients’ scores, based on the same visual analogue scales, with the actometer data was lower (*r* = 0.44) and non-significant. Not surprisingly, different investigators have used various terms to describe the motor activation, thereby revealing different underlying etiological concepts (Table 2), which center on the perceived syntonicity of the behavioral activation as one important dimension. The compulsive nature is seemingly more relevant than the absolute amount of physical activity (Adkins and Keel, 2005; Cook and Hausenblas, 2008). Adkins and Keel (2005) identified at least 31 different terms for unhealthy exercise.

**Table 2:** Terms used for describing motor activation in the acute stage of anorexia nervosa arranged according to insinuated syntonicity (Hebebrand et al., 2003).

Ego-syntonic	Activation and arousal, paradoxical liveliness, excessive vitality, abundance of physical energy
Neutral	Extensive exercise, intense athleticism, hyperactivity, over-activity, motor restlessness, diffuse restlessness
Ego-dystonic	Excessive exercise, exaggerated need for physical activity, compulsive exercising


There is currently no international consensus as to the operationalization of hyperactivity or motor restlessness in patients with AN. It has been proposed to specifically include “behavior(s) indicative of high energy expenditure” as a facultative diagnostic symptom in classification systems (Hebebrand and Bulik, 2011). The lack of a common standard substantially hampers the interpretation of published data. Hence, the split of a referred sample according to physical activity levels (Kostrzewa et al., 2013) may well render different associations than a comparison of severely hyperactive with non-hyperactive patients. Exercise in the eating disorder literature is generally characterized *quantitatively* in terms of frequency, duration, and intensity and *qualitatively* via features of compulsivity (Wyatt, 1997) including rigidity, increasing priority over other activities, being rule-bound, detailed record keeping, and feelings of guilt and anxiety over missed exercise sessions (Adkins and Keel, 2005; Noetel et al., 2017). A *third* dimension encompassing the motivation for exercise has also been proposed (Mond et al., 2006; Boyd et al., 2007). In patients with eating disorders, the term compensatory behavior describes the intention to counteract weight gain associated binge eating or to prevent weight gain (Lepage et al., 2008). Another classification is based on (a) excessive exercise (i.e., exceeding 6 h per day) and/or evocation of negative emotional states upon postponement of exercise, (b) high commitment to exercise despite adverse consequences for health or social contacts, and (c) a restlessness which entails a continuously active posture, fidgeting, or the inability to sit still (Kostrzewa et al., 2013).

Whereas the elevated physical activity may initially be experienced positively, it can turn into a behavior largely out of control. Because patients can resort to physical activity/exercise to intentionally lose weight or maintain a reduced body weight, the distinction between purposeful/intended and compulsive physical activity is at times fuzzy on an individual basis. Indeed, true exercise represents an intentional, planned, structured, and repetitive type of physical activity (Achamrah et al., 2016). However, there is no doubt that patients can perceive the urge to relentlessly exercise as beyond their control (see case report, Box 1). Patients should be specifically queried as to their perception of control of their physical activity. Their overall lack of experienced anxiety in light of their condition including excessive exercising may induce the observer to assume a more ego-syntonic quality of the respective behaviors.

**Box 1**

28 year old female with AN since age 15 with restricting and binge-eating/purging phases, BMI upon initial episode 14 kg/m^2^. Contestant at the national level for gymnastics prior to development of AN. After several treatment episodes partial stabilization for three years at BMI of ∼17.5 kg/m^2^.

During a trip to Africa relapse as a consequence of diarrhea and weight loss of 6 kg, development of extreme hyperactivity (walking briskly up to 10 h a day); subsequent further severe weight loss (BMI of 10 kg/m^2^); admission to the intensive care unit in pre-terminal condition; severe leukopenia.

With adequate intensive care rapid improvement of the general condition (BMI 13 kg/m^2^), but further increase of hyperactivity. High dosages of atypical neuroleptic medication and benzodiazepines without substantial effect on hyperactivity.

To provide guidance with respect to the controversy regarding the definition of clinically significant exercise, Noetel et al. (2017) conducted a Delphi study including 25 experts in adolescent AN to synthesize knowledge. The preferred term for unhealthy exercise in adolescents with AN was compulsive exercise. The panel did not consider a uniform cut-off as helpful for the definition of unhealthy exercise, instead the consideration of the exercise behavior in the context of the adolescent’s overall presentation and history was deemed relevant. A large portion of the endorsed items for unhealthy exercise were clinical markers of compulsivity such as distress associated with ceasing or delaying exercise, behavioral rigidity, and exercise performed despite negative health consequences.

From a psychopathological perspective, higher levels of anxiety, anhedonia, depression, inner restlessness (being anxious and jittery), irritability, somatization, obsessive thoughts, rigidity, restraint in emotional expression, perfectionism, high persistence, low novelty seeking, reward dependence, greater impulse control, obsessive-compulsive personality, and comorbid obsessive compulsive disorder have been associated with hyperactivity in AN (Davis et al., 1999; Davis and Woodside, 2002; Hebebrand et al., 2003; Holtkamp et al., 2004, 2006; Pike et al., 2008; Carrera et al., 2012; Sternheim et al., 2015; Achamrah et al., 2016). These associations may be causally related; for instance, anxiety may trigger hyperactivity or vice versa (Sternheim et al., 2015; Achamrah et al., 2016). Hyperactive patients are characterized by greater eating disorder psychopathology including more bulimic symptoms and a greater degree of body dissatisfaction and weight preoccupation (Hebebrand et al., 2003). Hyperactivity has been associated with both the restricting and binge eating/purging type of AN (Shroff et al., 2006; Dalle Grave et al., 2008).

A logistic regression analysis based on 153 inpatients with AN explained 31% of the variance in exercise status (excessive vs. non-excessive). Higher levels of dietary restraint, depression, and unexpectedly self-esteem predicted the exercise status as did, also unexpectedly, lower levels of obsessive-compulsive symptomatology and the restricting subtype of AN (Bewell-Weiss and Carter, 2010). The investigators point to the limitation that the results may have been distorted because the patients had not been able to exercise currently. Seemingly in contrast to the study of Bewell-Weiss and Carter (2010); Young et al. (2013) observed a positive association between excessive exercise and obsessive-compulsive symptoms in their literature review.

A high level of premorbid physical activity has been found to be associated both with AN per se and the hyperactivity of these patients in particular (Kron et al., 1978; Davis et al., 1994, 1997; Kostrzewa et al., 2013). According to Davis (1997), 60% of their interviewed hospitalized patients were competitive athletes prior to the onset of AN. In addition, 60% of patients predated sport or exercise to the onset of their eating disorder; the authors particularly noted that the eating disorder began after the patients had terminated participation in their sport. On average, the patients were more physically active than co-interviewed control subjects from early adolescence onward. This high level of activity may be an early indicator of the illness or alternatively and in our opinion more likely represents the upper end of inter-individual range of physical activity prior to the onset of the disorder. In a subsequent study, Davis (1997) showed that a significantly greater proportion of those patients who reported being highly physically active during childhood became excessive exercisers during their eating disorder as compared with their average/less active counterparts. Accordingly, elevated levels of premorbid physical activity would represent a risk factor for the development of hyperactivity upon onset of AN. In our clinical experience, adolescents who were engaged in competitive sports many months prior to the onset of AN frequently develop severe hyperactivity early in the course of their eating disorder.

At an epidemiological level, high activity levels have been linked to disordered eating behavior (Hebebrand et al., 2003). For example, the odds of ever being diagnosed with an eating disorder were 2.64 times higher for people who exercised more than 5 h per week in comparison to people with lower activity levels (Kostrzewa et al., 2013). We are unaware of a twin study related to hyperactivity in monozygotic twins concordant for AN; in general, the heritability estimate for physical activity levels is in the range of 0.5–0.6 (e.g., Kostrzewa et al., 2013).

In our experience, severe hyperactivity may stand in the way of the initiation of voluntary inpatient treatment, particularly in adults. Even after admission, both the treatment team and the respective patient may find it challenging to deal with hyperactivity within the constraints of a ward, thus, at times entailing a premature discharge. Hyperactivity has, however, also been associated with longer hospital stays and a chronic course (Solenberger, 2001; Achamrah et al., 2016).

Complications of excessive hyperactivity in AN include an increased risk of overuse injuries, bone fractures, and cardiovascular complications (Haddad et al., 1997; Dalle Grave, 2009). The prognostic significance of hyperactivity has been judged controversially. Some investigators have found evidence for a poor prognosis and an increased risk of relapse (Carter et al., 2004; Dalle Grave et al., 2008; Achamrah et al., 2016); in contrast, Kostrzewa et al. (2013) observed a better prognosis at the one year follow-up in adolescent patients who presented with high levels of physical activity.

In historic terms, clinicians dealing with hyperactive patients recommended bed rest to curtail the amount of energy spent for physical activity and to thus promote weight gain. However, it has become more and more evident that the treatment of patients with AN should include modules for physical activity. All experts, who participated in the aforementioned Delphi-Study (Noetel et al., 2017), recommended targeted interventions to adequately address unhealthy exercise, as opposed to relying on treatment approaches for AN in general. The potential benefits of such treatment programs using different types of modules have been summarized by Achamrah et al. (2016). However, to our knowledge, RCTs for the treatment of hyperactivity in patients with AN have not been performed. Particularly atypical neuroleptics (Mehler-Wex et al., 2008) are prescribed intermittently and off label to patients with severe hyperactivity.

Excessive exercise has not only been reported in patients with AN. The respective behaviors have also been observed in patients with Bulimia Nervosa and Eating Disorders Not Otherwise Specified (Davis et al., 1997; Renz et al., 2017). Furthermore, a link between exercise addiction and orthorexia nervosa has been reported (Rudolph, 2018). Among female athletes engaged in endurance sports, gymnastics, and ballet, a low energy intake and menstrual dysfunction including hypothalamic amenorrhea is rather prevalent (Misra, 2014). The highest prevalence is seemingly observed in patients with AN (Davis et al., 1997) despite single studies showing higher rates in patients with BN (Renz et al., 2017). It is important to point out that hyperactivity is not a common feature in famines nor was it common among the male participants of the Minnesota Starvation Study. However, some of the males reported symptoms indicative of restlessness (see Casper, 2018). In addition, among individuals who survived hunger episodes in World War II motor restlessness was also reported (Hebebrand et al., 2003).

Attempts to correlate serum leptin levels with activity levels measured via different methods in both adolescent and adult patients have provided mixed results (Exner et al., 2000; Holtkamp et al., 2006; Ehrlich et al., 2009; Kostrzewa et al., 2013; Nogueira et al., 2013; Stengel et al., 2017), potentially indicating that the threshold range for hyperactivity differs individually. Similar to the rat model, in which rats are no longer hyperactive prior to death due to starvation, exceedingly low leptin concentrations in patients may also reflect a terminal stage of the disorder, at which further weight loss would result in death. The inborn and virtually total lack of leptin in congenital leptin deficiency may explain why hyperactivity is not a symptom in the respective rare autosomal recessive condition. Accordingly, a non-linear relationship between circulating leptin levels and hyperactivity appears plausible (Holtkamp et al., 2006). Furthermore, hyperactivity in some patients may have a strong volitional component as the term drive for thinness suggests, which is not or only partially related to hypoleptinemia (Exner et al., 2000).

## Are Cognitions and Mood in Anorexia Nervosa Amenable to Treatment With Metreleptin?

In light of the proposed primary indication for treatment of severe hyperactivity in patients with AN, we believe that it is worthwhile to co-assess effects of metreleptin therapy on starvation-related cognitions. We hypothesize that they decrease upon treatment.

Patients with AN acknowledge that they reflect on food, eating, weight, and shape concerns for several hours a day. Upon inpatient treatment, the percentage of time spent on such thoughts will typically decrease; an adolescent patient may state that 90% of her time awake is spent on such thoughts during her first week of treatment, whereas 3 months later, upon achievement of her target weight, this percentage has decreased to 40%. Some patients state that these cognitions are bothersome, other patients experience them as ego-syntonic. During treatment, both clinicians and relatives can usually observe an increasing loosening of this apparent rigidity and obsession with such thoughts accompanied by a greater openness and willingness to dwell on other personal and emotional issues. Whereas both the therapeutic setting and weight gain may underlie this improvement, the realimentation process presumably plays the major role. Because starvation induces a pre-occupation with food and an altered eating behavior (Table 1), these cognitions should especially be reduced by restitution of a body weight in the healthy range. Importantly, as key endocrine signals underlie this process, the application (or reduction) of such hormones/neuropeptides and metreleptin in particular may also entail a reduction of such cognitions and behaviors. We hypothesize that specific cognitions and/or affective symptoms associated with hyperactivity in patients with AN also decrease, if hyperactivity in itself can successfully be treated with metreleptin.

The potential impact of leptin on depression like behavior was indirectly substantiated in mouse models with selective LEPR ablation in the adult hippocampus (Guo et al., 2013). It is thus carefully and more generally hypothesized that metreleptin may entail positive effects in depression (Paz-Filho et al., 2015). Irritability and a depressed mood represent common symptoms in patients with AN. In the Minnesota Starvation Experiment, these symptoms became more pronounced in some men during the realimentation period (Guetzkow and Bowman, 1946). Similarly, in patients with AN, mood may deteriorate during the refeeding phase; some patients appear anxious to overeat and to lose control of their eating behavior, which may evolve into binge eating. On average, however, mood and eating behavior clearly improve during refeeding (Carter et al., 2004), which may in part be the result of leptin-induced mood improvement. Accordingly, we hypothesize that a depressed mood improves in patients with AN who are treated with metreleptin. Mood should thus be co-assessed.

## Anorexia Nervosa, Leptin, and Reward

The striking behavior of patients with AN and their restriction of food intake to the extent of self-starvation in particular have prompted researchers to come up with potential explanations, which more recently have focused on the reward system including the underlying brain regions, pathways, and neurotransmitter systems, among which the DA system figures prominently. A genetic predisposition (including female sex) along with specific temperamental and personality features, hormonal factors, and brain maturation processes occurring during puberty and adolescence may result in a high vulnerability to AN, if food restriction is initiated and pursued to an extent that starvation related changes set in. A twin study has pointed to an altered reward reactivity as a potential risk endophenotype for eating disorders (Kanakam et al., 2017). Speculatively, the predisposing genotype may render carriers to more readily get entrapped in the cognitions and behaviors characteristic of AN, if food restriction, subsequent weight loss, and the adaptation to starvation are initiated in adolescence/early adulthood irrespective of the reason/mechanism underlying the initially reduced food intake.

Several investigators have argued that food restriction and in due course starvation may in some way sensitize the reward system, thus, contributing to or even representing an essential feature for the relentless pursuit of thinness in patients with AN (Keating, 2010). Psychological explanations include the alleviation of anxiety and a dysphoric or anhedonic mood state (Davis and Woodside, 2002); accordingly, the food restriction including the sensation of hunger serves to regulate emotions. The psychological effects of food restriction are embedded in the profound physiological and biological alterations of initial and later prolonged starvation, for which hypoleptinemia acts as a crucial endocrine signal.

Patients with AN seemingly have disturbances in the processing of naturally rewarding stimuli, importantly including food. The processing of food reward is complex and modulated by cognitive, emotional, and biologic factors that involve learned behaviors and genetic predisposition (Frank, 2011). Evidence from taste reward tasks indicates that patients do not differ from healthy controls with respect to the hedonic properties of taste-reward; their “liking” of foods is not altered in comparison to controls. Instead “wanting” in patients for the preference for stimuli such as high caloric foods may be reduced as a result of the fear of weight gain (Keating, 2010).

All currently investigated neuropeptides and hormones tightly linked to appetite and body weight regulation have been found to be up- or downregulated in a manner consistent with starvation (Stoving et al., 2001) in patients with acute AN. Apart from the reduction of serum or cerebrospinal leptin levels (Hebebrand et al., 1995, 1997; Grinspoon et al., 1996; Mantzoros et al., 1997), examples include the elevations of neuropeptide Y (Kaye et al., 1990) and ghrelin (Ariyasu et al., 2001; Otto et al., 2001). Accordingly, the neuroendocrine adaptation to starvation is seemingly not impaired in patients. The respective abnormalities do not persist upon recovery, suggesting that primary disturbances of appetite regulation are not at the core of AN (Stoving et al., 1999, 2001; Eddy et al., 2015); they may, however, contribute to a vicious circle upon initiation of starvation (Stoving et al., 1999).

We specifically postulate that the hypoleptinemia-induced adaptation of the central nervous system contributes to the initiation of such a vicious circle in individuals predisposed to develop AN. The role of leptin in the reward system has been pointed out above including the link to the DA system. Leptin has extra-hypothalamic effects (van der Plasse et al., 2015) in the regulation of food intake, reversibly altering neural structure and function, and modulating plasticity-dependent brain physiology in response to food cues (Baicy et al., 2007; Paz-Filho, 2016). An increase in gray matter concentration was observed in anterior cingulate gyrus, parietal lobe, and medial cerebellum of adults with congenital leptin deficiency, who were treated with metreleptin for 18 months (Paz-Filho et al., 2015; Paz-Filho, 2016). The increase in the medial cerebellum was directly due to metreleptin treatment. In functional studies performed with patients with congenital leptin deficiency, metreleptin treatment reduced activation of regions linked to hunger including insula, parietal and temporal cortex, and enhanced activation of regions linked to inhibition and satiety (prefrontal cortex), as well as the posterior lobe of the cerebellum. Somewhat divergent from these results, Frank et al. (2011) and Paz-Filho (2016) observed changes in homeostatic (hypothalamus) and reward-related brain areas [striatum, orbitofrontal cortex (OFC), substantia nigra/VTA, amygdala] 3 days and 6 months after initiation of metreleptin therapy in an adolescent with congenital leptin deficiency. In a follow-up study conducted after 12 and 24 months, metreleptin therapy was associated with activation changes in homeostatic, hedonic, and frontal control regions (Frank et al., 2013).

In AN, it would appear likely that the detected higher connectivity between the OFC and the hypothalamus on the left and the lower connectivity between basolateral nucleus of the amygdala and hypothalamus on the right (Frank et al., 2016) represent a structural adaptation to starvation in patients with AN. The involvement of the hypothalamus is in line with its key role in appetite and body weight regulation (Frank et al., 2016) and indicative of an altered connectivity which extends beyond the reward system. With respect to this system, Frank et al. (2016) detected a bilaterally higher connectivity between insula, frontal cortex, and ventral striatum in eating disordered patients. The investigators discussed that the effective network connectivity from anterior cingulate to ventral striatum and to the hypothalamus may provide a possible biological correlate for the hypothesis that patients with eating disorders are able to override homeostatic signals.

The critical role of the dorsal striatum in the establishment and expression of action control and learned automatic behaviors was supported in a study based on functional magnetic resonance imaging (fMRI) to compare blood oxygen level-dependent (BOLD) activity among women with acute AN with that of healthy female controls upon use of a food choice task that captures the restrictive caloric intake (Foerde et al., 2015). The persistent, maladaptive food choice in AN is subserved by fronto-striatal networks that are crucial for the development of habitual behavior and maladaptive human behaviors.

Giel et al. (2013) compared the reward of physical activity in patients with AN, athletes, and controls using eye-tracking. Both patients and athletes rated active stimuli as more pleasant as compared to non-athletes, and patients rated passive stimuli as more unpleasant than both control groups. A strong correlation between reward sensitivity and attentional orienting was observed among the patients; furthermore, the amount of individually performed physical activity and pleasantness ratings of active stimuli were associated with both attentional orienting and engagement. The negative appraisal of physical inactivity might promote physical activity and hyperactivity in patients with AN. In the corresponding fMRI study, both patients with AN and athletes revealed a hyperactivation in the somatosensory cortex in response to physical activity stimuli, suggesting that these stimuli are perceived as more rewarding than in non-athletic controls. Physically inactive stimuli resulted in an increased response inhibition associated activity in the patients with AN only, whereas physically active stimuli resulted in a reduced activity in the cerebellum (Kullmann et al., 2014).

Based on the concept of stress induced reward linked to illness behaviors (Bergh and Sodersten, 1996), Keating (2010) formulated the reward contamination theory, according to which patients experience otherwise rewarding stimuli (e.g., food) as punishing, and vice versa otherwise punishing (or aversive) stimuli (e.g., self-starvation, excessive physical activity, emaciated body image) as rewarding. It is unclear whether reward contamination begins as a result of an aversion to food or increasing social/cognitive goals of thinness. The term perverse reward has also been coined (Park et al., 2014) to characterize the pursuit of thinness and compulsive engagement in extreme dietary restraint, often in combination with over exercising. Perverse reward is considered to be accentuated in line with starvation; the clinical problems inherent to treatment of AN are due to the compulsivity associated with perverse reward. Because food restriction amplifies reward processes, the self-enforced abstinence in AN may entail that food becomes more rewarding, thereby explaining some cognitions (e.g., constant thoughts of food) and behaviors (e.g., ritualistic eating behavior) that apply to both patients with AN and subjects exposed to starvation (Park et al., 2014).

The anterior cingulate cortex, the insula, the entire fronto-striatal neurocircuit, and the default mode network have been proposed to represent key loci for reward-contamination/perverse reward (Keating, 2010; Park et al., 2014). The ventral striatum is involved in coding the pleasure of a reward. Motivational salience, which too has been linked to the ventral striatum, is defined as the process through which a stimulus is converted from a neutral representation into an attractive and wanted incentive that a person will work to acquire (“wanting”) (Park et al., 2014). The dorsolateral prefrontal cortex has been suggested to modulate striatal activity that underlies the approach or avoidance of food (Kaye et al., 2009). Accordingly, an increased activation in this area in response to aversive food stimuli in recovered patients with AN may, therefore, represent an enhanced attempt to minimize exposure to such stimuli (Kaye et al., 2009; Park et al., 2014). Increased functional connectivity between the default mode network and both the precuneus and the dorsolateral prefrontal cortex in recovered patients suggests that resting state networks involving self-referential processing and cognitive control may be dysfunctional in AN (Park et al., 2014). Park et al. (2014) conclude that “neuroimaging studies suggest that altered eating may be a consequence of aberrant reward processing in the context of heightened cognitive control processes and interoceptive deficits, leading to poor awareness of homeostatic needs”; these mechanisms promote the compulsive behaviors observed in patients.

Evidence implicates the DA system in the aberrant reward processing of patients with AN (for links between the DA system and leptin see above). Keating (2010) proposed that an upregulation of the stress HPA and the dopamine system are linked to the self-starvation and excessive exercise of patients with AN, thereby entailing that the behaviors reinforce the illness and emaciation. Whereas the upregulation of the HPA axis represents a consequence of starvation, the persistence of abnormalities of the DA system in recovered patients was deemed as suggestive of a trait marker. Thus, an increased D2 and D3 receptor binding in the reward linked antero-ventral striatum was identified in recovered patients (Kaye et al., 1999; Frank et al., 2005). Because the binding potential of [(11)C]raclopride used to assess dopamine D2/D3 receptor binding was positively related to harm avoidance in the dorsal caudate and dorsal putamen, decreased intra-synaptic dopamine concentration or increased D2/D3 receptor density or affinity may be associated with AN and contribute to the characteristic harm avoidance or increased physical activity found in AN. Indeed, the finding of reduced concentrations of dopamine metabolites in the cerebrospinal fluid of recovered individuals relative to controls (Kaye et al., 1999) supports this hypothesis. An altered DA activity in AN is further suggested by the observation of an increased eye-blink compared with controls (Barbato et al., 2006). A comparative study based on a taste reward task in patients with AN, subjects with obesity and controls suggested that in obesity dopamine related pathways may be less sensitive, whereas the opposite holds true for AN (Frank et al., 2012).

Based on an elevated prediction error signal in a sample of patients with AN, food restriction and weight loss were postulated to be associated with sensitization of the dopamine system, potentially in order to stimulate food approach. The elevated prediction error response may reflect increased harm avoidance, because the biological mechanism to seek food is not compatible with the high drive for thinness and body dissatisfaction, entailing a conflict between food approach mechanisms and cognitive-emotional processes that oppose eating. Prediction error activation may then become part of a fear-driven mechanism that includes the ventral striatum to override homeostatic signals from the hypothalamus (Frank et al., 2018).

**FIGURE 6:**
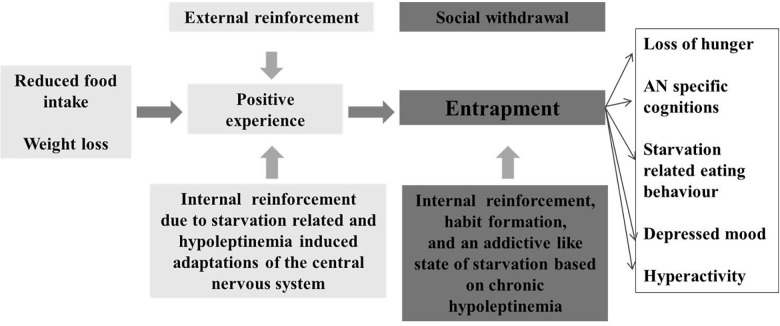
Model for the entrapment of patients with anorexia nervosa due to an addictive like state of starvation, which evolves after initial food restriction and entails an internal reinforcement.

In conclusion, brain-imaging studies of brain reward function in AN are strongly indicative of brain reward system alterations. Whereas some evidence points to primary disturbances, persistent longer term starvation-induced effects cannot be excluded (King et al., 2018). Thus, several rodent studies have revealed inductions of alterations of brain reward pathways in relationship to energy intake; in essence, food restriction sensitizes, whereas excessive food intake desensitizes reward pathways (see Frank et al., 2012). A psychological explanation for the development and persistence of AN postulates that a reluctance to gain weight may lead to an aversive appraisal of food- and taste-related stimuli. Accordingly, cues compatible with this aberrant mode of thinking become rewarding; attribution of motivational salience to these cues promotes anorectic behaviors and elicit the anorectic “habit” (O’Hara et al., 2015). We propose that adaptation of the central nervous system to starvation (crucially initiated via hypoleptinemia) in itself may represent a strong determinant for the anorectic “habit” (Figure 6). Both the psychological (O’Hara et al., 2015) and the endocrine induced starvation hypotheses focus on the reward system and postulate an overlap between AN and addiction.

## The Need for Clinical Trials

We have perceived the need for the initiation of clinical trials to assess the potential merits of metreleptin in the treatment of patients with AN for several years (Exner et al., 2000; Hebebrand and Albayrak, 2012). However, our past contacts with representatives of the companies with the rights on metreleptin proved unsuccessful with respect to the initiation of a clinical trial. The elucidation of the central pathways operative in appetite and body weight regulation has enabled the evaluation of potential novel treatments of AN. Recently, promising results were achieved in a 4 week long RCT encompassing 22 patients with AN with the ghrelin agonist relamorelin, which led to a shorter gastric emptying time and a trend toward greater weight gain (Fazeli et al., 2018). The fact that three patients stopped the use of relamorelin due to increased feelings of hunger, underscores problems inherent to the use of an orexigenic agent to treat this eating disorder.

The recent FDA (Meehan et al., 2016) and EMA (EMA, 2018) approvals of metreleptin for the treatment of lipodystrophy render the initiation of trials into other hypoleptinemic disorders possible with AN ranking prominently among these. Instead of increasing the already elevated orexigenic drive, recombinant leptin could ameliorate the starvation-related symptomatology (Table 1) in patients in light of its key role in the endocrine-induced adaptation of the organism to starvation. Thus, treatment could entail a (partial) resolution of secondary symptoms of this eating disorder leading to a “residual” symptomatology with an ensuing preponderance of the primary cognitions and, thus, a (partial) clinical dissection of the intertwined primary and starvation-related symptoms of AN (Hebebrand and Bulik, 2011). In weight restored patients, metreleptin therapy may also speed up the recovery of the reproductive axis with potentially beneficial effects on bone health. Initially, we suggest to focus on severe AN associated hyperactivity (Table 3). Such a trial would allow the assessment of other potentially beneficial effects on the psychopathology of these patients among which we particularly suggest that food related cognitions would decrease during therapy.

**Table 3:** Potential indications for clinical trials to assess the effects of metreleptin therapy for patients with anorexia nervosa (AN).

Primary outcome	Remarks
Physical activity level	A priori highest probability of efficacy; inclusion of not severely emaciated patients ≥18 years, who can provide informed consent. Co-assessment of inner restlessness, anxiety, and obsessive thoughts related to exercise outcomes (see first column). Co-assessment of other psychopathological features (e.g., other eating disorder specific cognitions) and multiple safety parameters. Clear *a priori* determination of dosage increments, maximal dose and lengths of treatment for both unsuccessful and successful treatment. *A priori* definition of successful treatment. Precautions and close monitoring required to avoid metreleptin-induced weight loss
Self- (and clinician-)rated quantity (e.g., percent of time) of (a) starvation-related cognitions including food and eating and body weight-related ruminations, (b) abnormal eating behaviors, (c) mood/anxiety/obsessions and compulsions, and (d) reward sensitivity	Initial evidence for an effect on such cognitions/behaviors/mood to be obtained in trials for hyperactivity; co-assessment of multiple safety parameters. Initial inclusion of adult patients only, patients <18 years can be included, if treatment-related serious adverse events do not occur (patient/parental consent required)
BMI at end of study to assess halt of progression of early stage AN	Patients of any age whose initial weight loss began less than ≤4 months ago
a) BMI at end of study to assess effectiveness in relapse prevention b) Weeks until first relapse	Patients of any age who initially recovered from first episode of weight loss and have maintained their target weight for at least 2 months with subsequent loss of 2 kg as an indicator for a relapse Patients of any age who have achieved their target weight
Menarche or resumption of menses	Patients aged ≥14 years who have maintained their target weight for 6 months; requirement of a relative fat mass within the normal range
Bone density	Treatment of adult patients with osteoporosis and/or fractures


*The rank order provides guidance as to the temporal enrollment of the respective trials.*

In more general terms, metreleptin may reduce the reward inherent to the process of the endocrine initiation of starvation in thus predisposed subjects. The process of starvation is somehow intricately linked to AN (Steinglass and Foerde, 2018), thus, driving patients to “perversely” (or paradoxically) pursue the restricted energy intake. We hypothesize that the obsessive and perverse urge to continue to maintain (or even increase) the reduced energy intake may represent an addiction like state (Figure 5). If this hypothesis is correct, a patient who is in the initial stages of AN may profit from early treatment with metreleptin. The induced halt and reversal of the endocrine adaptation to starvation would accordingly neutralize the addictive state so that patients no longer experience reward via a continued reduction of energy intake. In patients with longer standing AN, metreleptin may also help to reduce the reward associated with starvation, thus alleviating the entrapment in the disorder. The mere reduction of the obsessive thoughts of food could in theory entail that the patient no longer perceives her restricted food intake as a major accomplishment in the sense that is worthwhile to fully engage in.

## Potential Side Effects and Risks of Treatment with Recombinant Leptin

As with any other treatment, potential side effects warrant a crucial reflection (Table 4; a complete overview is beyond the scope of this review). Treatment with metreleptin can provoke the occurrence of several adverse events. The most frequent adverse events are development of anti-metreleptin antibodies, headache, nausea, hypoglycemia, decreased weight, and increased risk of infections (Ajluni et al., 2016; Brown et al., 2018). Table 4 lists the risks and side effects of the MYALEPT^®^ information package to be used upon metreleptin treatment. However, additional adverse events such as urinary tract infection (Ajluni et al., 2016) and hair loss (Chan et al., 2011) have been reported.

**Table 4:** Risks and adverse reactions to metreleptin (results from two studies among women with hypothalamic amenorrhea and package insert information of Myalept^®^ for the United States, Myalepta for the European Union). This information is supplied without liability.

Welt et al., 2004^×^	“There appeared to be no adverse effects (including injection-site reactions) during therapy with r-metHuLeptin. Subjects reported a qualitative decrease in appetite, primarily during the third month, but otherwise felt well”	
Chou et al., 2011γ; Sienkiewicz et al., 2011	Withdrawals *n* = 2 (injection site reaction and weight loss)	
	Mild injection site reactions *n* = 2	
	Non-neutralizing antileptin antibodies	
	No other clinically adverse events	
**Myalept^®^ Package insert**		
Risks+	Development of antibodies that neutralize endogenous leptin and/or MYALEPT	
	Lymphoma	
	Hypoglycemia with concomitant use with insulin and insulin secretagogues	
	Autoimmunity	
	Hypersensitivity	
	Benzyl alcohol toxicity	
Adverse reactions^∗^ (*N* = 48)	Headache, hypoglycemia, decreased weight	each *N* = 6
	Abdominal pain	each *N* = 5
	Arthralgia, dizziness, ear infection, fatigue, nausea, ovarian cyst, upper respiratory tract infection	each *N* = 4
	Anemia, back pain, diarrhea, *paresthesia*, proteinuria, pyrexia	each *N* = 3


*^×^Incidence in female study participants (*n* = 8) with hypothalamic amenorrhea (Welt et al., 2004). γ Incidence in female study participants (*n* = 11) with hypothalamic amenorrhea (Chou et al., 2011; Sienkiewicz et al., 2011). +As stated in the Instructions for Use, Manufactured for: Aegerion Pharmaceuticals, Inc. Cambridge, MA 02142 MYALEPT is a registered trademark of Aegerion Pharmaceuticals, Inc. PN 4086-00 Issued August 2015 MYA/US/058 10/15. ^∗^Incidence in patients with generalized lipodystrophy receiving MYALEPT in an open-label, single-arm study.*

The potential weight loss due to metreleptin therapy in patients with AN would warrant special attention and consideration in clinical trials. Among women with hypothalamic amenorrhea (mean BMI 20.5 ± 2.0 kg/m^2^ at baseline) under leptin treatment, body weight, and body fat decreased, especially during month 3 with higher dosages of leptin (body weight: baseline: 54.7 ± 4.5 kg, month 1: 54.1 ± 4.3 kg, month 2: 54.0 ± 3.6 kg, month 3: 52.2 ± 3.5 kg). No adverse reactions due to metreleptin treatment were observed (Welt et al., 2004). In another study among women with hypothalamic amenorrhea, one out of 11 participants stopped treatment due to persistent weight loss during the first phase, while a second participant withdraw during the second phase. Apart from three participants with injection site reactions, no other adverse events due to the metreleptin treatment were seen (Chou et al., 2011; Sienkiewicz et al., 2011).

The partial metreleptin-induced resolution of the adaptation to starvation should entail an increased sympathomimetic and a reduced parasympathetic tone. As such, body temperature, heart rate, and blood pressure would likely increase in organisms weakened by starvation, thus, necessitating a close monitoring. Obviously, these alterations would entail an elevated resting energy expenditure, which might need to be compensated via an increased energy intake. The initiation of treatment with metreleptin would appear grossly negligent if the patient *a priori* does not agree to increase his/her energy intake accordingly. The overall goal of weight restoration resulting in the synthesis of a sufficient amount of endogenous leptin must be maintained, thus entailing that metreleptin is an intermittent therapy only.

Because many patients with AN may no longer perceive hunger, the elevated leptin signal should not entail a reduced appetite, and, as a consequence an even further reduced energy intake. If on the other hand, the patient experiences hunger, the loss of this sensation via leptin treatment may speculatively entail that the patient no longer is motivated by her ability to overcome hunger; the strong incentive to continue to stick to a reduced energy intake may no longer be operative. Irrespective of these considerations, we again stress the need to monitor energy intake during treatment with human recombinant leptin to ensure that the treatment does not entail weight loss.

Temporal aspects merit consideration. Whereas the treatment of rats after 5 days of food restriction in the SIH model entails a rapid and pronounced reduction of RWA (Exner et al., 2000), the hypothesized effect on AN related hyperactivity may require more time in humans. Obviously, the treatment goal of restoration of menstruation may require several months for the reproductive axis to recover, even if leptin serum concentrations are boosted to the normal range via application of metreleptin. All endocrine/physiological barriers to resumption need to be overcome including for instance the renewed growth of the ovaries. In this context, it deserves to be pointed out that among leptin deficient individuals, who harbor relevant mutations in the leptin gene, antibody production against leptin has been observed. As a rule, a transient halt in treatment enabled renewed and effective treatment with recombinant leptin (Farooqi et al., 2002; Frank et al., 2013); to our knowledge, no serious side effects have been observed in patients with inborn leptin deficiency despite treatment lasting for several years.

## Conclusion

In the light of the problems patients and their families, friends, and partners struggle with, physicians and the pharmaceutical industry have the obligation to test novel treatments for AN. Rodent data and more importantly, successful small scaled trials for both hypothalamic amenorrhea and osteoporosis (Welt et al., 2004; Sienkiewicz et al., 2011) in young and lean females suggest that the risks inherent to treatment with leptin are manageable, if adequate safety precautions are met. Metreleptin might prove to be effective in treating several starvation induced symptoms of AN including hyperactivity, eating disorder-related ruminations, and in addition impairments in bone metabolism, reproductive function, and hematopoiesis. Finally, if the adaptation to starvation is rewarding or even addictive, patients may be able to more readily overcome their eating disorder. If metreleptin proves successful in the management of patients with AN, psychotherapy and other treatment modalities will benefit from an incorporation of the respective findings. Importantly, it remains to be seen what percentage of patients would be willing to be treated with metreleptin (Hebebrand et al., 2019, in press).

## Author Contributions

All authors wrote many chapters, added figures, and reviewed the whole manuscript.

## Conflict of Interest Statement

The authors declare that the research was conducted in the absence of any commercial or financial relationships that could be construed as a potential conflict of interest.

## Abbreviations

ABA: activity-based anorexia
AgRP: agouti-related protein
AN: anorexia nervosa
BMI: body mass index
DAT KO: dopamine-transporter knockout
DNA: deoxyribonucleic acid
DSM-5: Diagnostic and Statistical Manual of Mental Disorders, 5th Edition
EMA: European Medicines Agency
FDA: Food and Drug Administration
FOXO1: Forkhead box protein O1
FSH: follicle-stimulating hormone
HPA: hypothalamo-pituitary axis
ICD-11: International Classification of Diseases – 11
ICV: intracerebroventricular
IRS: insulin receptor substrate
JAK: Janus kinase
KATP: ATP-sensitive potassium channel
LEPR: leptin receptor
LH: luteinizing hormone
LRb: long form of leptin receptor
PI3K: phosphatidylinositide 3-kinase
POMC: proopiomelanocortin
PTB1B: protein tyrosine phosphatase 1B
RCT: randomized controlled trial
r-metHuLeptin: recombinant methionyl human leptin
RWA: running wheel activity
SH2: Src-homology 2
SHP2: Src homology phosphatase 2
SIH: starvation-induced hyperactivity
SOCS3: suppressor of cytokine signaling 3
STAT3: signal-transducer activator transcription 3
TRP: tyrosinase-related protein
VTA: ventral tegmental area
